# A Feasibility Study of Literature-Guided HRV Stratification Using Large Language Models

**DOI:** 10.3390/diagnostics16040540

**Published:** 2026-02-11

**Authors:** Tien-Yu Hsu, Gau-Jun Tang, Cheng-Han Wu, Jen-Tin Lee, Terry B. J. Kuo

**Affiliations:** 1 Institute of Brain Science, National Yang Ming Chiao Tung University, Taipei 11221, Taiwan; 2 Sleep Research Center, National Yang Ming Chiao Tung University, Taipei 112304, Taiwan; 3 Institute of Hospital and Health Care Administration, National Yang Ming Chiao Tung University, Taipei 112304, Taiwan; 4 Department of Information Management, National Taipei University of Nursing and Health Sciences, Taipei 112303, Taiwan; 5 Department of Otolaryngology, Auditory Medical Center, Cheng Hsin General Hospital, Taipei 11220, Taiwan; 6 Brain Research Center, National Yang Ming Chiao Tung University, Taipei 112304, Taiwan; 7 Center for Mind and Brain Medicine, Tsaotun Psychiatric Center, Ministry of Health and Welfare, Nantou 542019, Taiwan

**Keywords:** cardiovascular, cerebrovascular, heart rate variability, clinical decision support system, large language models, literature mining

## Abstract

**Background:** Heart rate variability (HRV) is a valuable indicator for assessing vascular health, but keeping clinical decision support systems (CDSSs) aligned with the rapidly evolving literature remains challenging. This study aimed to develop an LLM-assisted literature synthesis framework to support transparent HRV-based risk stratification, enabling systematic extraction and organization of HRV evidence from published studies. **Methods:** An LLM-driven framework was developed to extract HRV parameters from 140 medical abstracts. The system simulated step-by-step human reasoning to identify key HRV indicators and group patient data using predefined statistical thresholds derived from the literature. System performance was evaluated using ECG-derived HRV features as a feasibility evaluation of literature-guided HRV classification. **Results:** The proposed framework demonstrated an accuracy of 86% in literature-guided HRV classification, with a sensitivity of 81% and a specificity of 87%. Compared with traditional machine learning approaches, the LLM-assisted system provided transparent, literature-grounded reasoning and could be readily updated as new studies became available. **Conclusions:** Large language models can support evidence-guided parameter selection and feasibility-level HRV-based risk stratification, rather than serving as predictive classifiers. This approach reduces manual effort, enhances transparency, and addresses common “black box” concerns associated with AI-assisted CDSS development in clinical practice.

## 1. Introduction

Cardiovascular and cerebrovascular diseases are the leading cause of death and disability worldwide [1,2]. Predicting cardiovascular and cerebrovascular events has always been an important topic because it allows for early treatment to prevent progression of the disease to an irreversible stage [3]. One method for predicting such events is heart rate variability (HRV), which refers to the variation in time intervals between successive heartbeats. Studies have reported that HRV reflects the autonomic nervous system, including the sympathetic and parasympathetic nerves [4,5,6,7] that regulate multiple organs in the body, making HRV a predictor of organ condition [8,9]. Meta-analyses confirm HRV’s strong prognostic value for cardiovascular outcomes. Fang et al. analyzed 28 cohort studies of cardiovascular disease patients and found low HRV associated with doubled all-cause mortality risk and 46% higher cardiovascular event risk, across time and frequency domains [10]. Similarly, a comprehensive review of 32 studies and 38,008 participants showed reduced HRV metrics predict elevated all-cause and cardiac mortality risks, independent of demographics, population type, or adjustments [11]. Since 1997, our team has been using arterial blood pressure variability and HRV to predict Intensive Care Unit (ICU) patient prognosis, and we have found that the frequency domain analysis parameter of HRV could be used as a predictor of survival [12]. In 2014, Paolo Melillo et al. created a model using retrospective data from the Holter database to review electrocardiograms (ECGs) of patients with high blood pressure and track whether these patients had a cardiovascular and cerebrovascular event in one year. By using HRV parameters, their model, which employed classical machine learning methods, achieved sensitivity and specificity rates of 71.4% and 87.8%, respectively [13]. A retrospective study [11] found that HRV could be a non-specific predictor of mortality, and the results of this study revealed a significant negative correlation between HRV measurements and mortality. Based on these results, they recommended reporting a minimum set of HRV parameters in all future studies, including the standard deviation of all normal-to-normal intervals (SDNN), square root of the mean of the square of successive NN intervals (rMSSD), power in the low-frequency range (LF), power in the high-frequency range (HF), total power (TP) over 5 min recordings, and power in the very-low-frequency range (VLF) over 24 h recordings.

The literature on HRV in the human body is extensive because HRV measurement is a non-invasive, cost-effective, and rapid examination method. According to PubMed, over 1000 HRV-related studies have been published annually since 2018. Recent studies have applied HRV and heart rate (HR) analysis beyond cardiovascular health, using it to examine autonomic nervous system (ANS) responses in various psychological and neurological domains. For instance, researchers have investigated HRV in fear conditioning and PTSD [14,15], error-related autonomic regulation [16], and broader brain–body interactions in psychiatric and neurological diseases [17]. These studies highlight the growing diversity and complexity of HRV applications, further contributing to the challenge of staying current with evolving evidence. Nevertheless, doctors struggle to keep up with this abundance of research owing to time constraints. On average, general practitioners can only dedicate 4.6 h per week to learning new specialties, which makes it challenging for them to stay updated on HRV advancements [18]. Currently, the HRV Clinical Decision Support System (CDSS) database, which was built by collating data from past studies, is used in clinical settings. However, this database has not been updated with new research findings. Consequently, the interpretation of clinical HRV results remains highly dependent on the knowledge and experience of physicians. Physicians face an overwhelming volume of literature, and traditional decision support systems are not designed to keep pace with new findings.

In this context, advances in natural language processing (NLP) and large language models (LLMs) offer a promising solution. NLP is the technology that allows computers to interpret human written data, and has evolved rapidly in recent years. In 2016, International Business Machines Corporation released Watson for Oncology, which applied traditional NLP techniques to analyze medical literature and assist physicians in organizing data and supporting decisions [19,20,21]. Unlike conventional machine learning methods that rely on explicit features for classification, NLP-based systems mimic aspects of human learning. Key advancements in language modeling began with Radford et al.’s generative pre-training model in 2018, which used large volumes of unlabeled data for self-supervised pre-training [22]. This was followed by Devlin et al.’s bidirectional encoder representations from transformers (BERT) in 2019, which significantly influenced the development of LLMs [23]. LLMs are capable of zero-shot learning—performing tasks without specific fine-tuning. For instance, OpenAI’s GPT-3.5, released in 2021 with 175 billion parameters, can summarize articles and generate structured outputs like tables [24,25,26].

While LLMs have demonstrated strong capabilities in summarizing and organizing large volumes of biomedical text, their effective use in clinical decision support systems depends heavily on how reasoning and knowledge integration are structured at the application level. In many existing medical NLP applications, LLMs are primarily employed in zero-shot or surface-level extraction settings, which limits their ability to support systematic, multi-step decision-making workflows required for quantitative interpretation and evidence synthesis. Recent methodological advances have focused on designing prompting and task-decomposition strategies that better align LLM outputs with structured reasoning processes. Wei et al. introduced the chain-of-thought (COT) prompting method, which improved reasoning performance by providing step-by-step exemplars [27]. Building on the COT, self-consistency was developed to enhance reliability by generating multiple reasoning paths and aggregating consistent answers [28]. Zhou et al. further proposed the least-to-most strategy, which decomposes complex tasks into sequential sub-problems, improving the model’s ability to handle intricate queries [29]. This strategy operates in two phases: decomposition and stepwise problem-solving, often combined with self-consistency to increase robustness. Although such approaches improve logical reasoning in mathematical contexts, most applications of LLMs in medicine still rely on zero-shot strategies rather than explicit reasoning chains [30,31].

Cardiovascular and cerebrovascular diseases remain leading causes of death globally, underscoring the importance of early risk identification. Heart rate variability (HRV) is a well-established, non-invasive physiological biomarker reflecting autonomic nervous system regulation, and has been shown to provide prognostic information for cardiovascular and cerebrovascular conditions. However, integrating the latest HRV findings into clinical decision support systems (CDSSs) remains challenging due to the volume and complexity of emerging literature. This study explores whether large language models (LLMs) can assist in automating the extraction of clinically relevant HRV parameters from recent research. By supporting abstract screening and structured literature analysis, the LLM identifies frequently reported HRV features linked to vascular events. These features are then tested on public ECG data for predictive utility. Our approach combines the interpretability of evidence-based parameter selection with the scalability of NLP tools, offering a framework to enhance CDSS performance with minimal human effort. This LLM-guided method may help maintain up-to-date, transparent, and clinically relevant decision support in real-world settings.

Importantly, this study does not aim to propose or validate a new predictive algorithm for cardiovascular or cerebrovascular events, nor to position large language models as alternatives to established statistical or machine learning-based prediction methods. Instead, our focus is on evaluating the feasibility of using LLM-assisted literature synthesis as a supporting tool for clinical decision support systems. Specifically, we investigate whether LLMs can facilitate the systematic extraction and aggregation of frequently reported, clinically interpretable HRV parameters from recent literature, thereby guiding transparent parameter selection and subsequent simple statistical risk stratification. By positioning LLMs as an auxiliary knowledge integration mechanism rather than a predictive engine, this work emphasizes interpretability, reproducibility, and ease of clinical adoption, while minimizing methodological complexity.

## 2. Materials and Methods

To operationalize this framework, we implemented a two-stream methodological pipeline that integrates automated literature analysis with conventional physiological signal processing. The study workflow consists of five major stages: (1) systematic literature retrieval and structuring via the LLM, (2) evidence aggregation based on the frequency and directionality of reported HRV associations, (3) literature-guided prioritization of HRV parameters using a structured prompting strategy, (4) HRV feature extraction from real-world ECG data, and (5) outcome classification and evaluation. This integrated design aims to simulate is intended to mirror conventional human literature-synthesis and data-analysis workflows while preserving the scalability and reproducibility advantages of an automated system. Throughout this pipeline, the LLM primarily functioned as a tool for literature structuring and evidence aggregation within a broader, rule-based analytical framework.

### 2.1. Dataset

**Figure 1:**
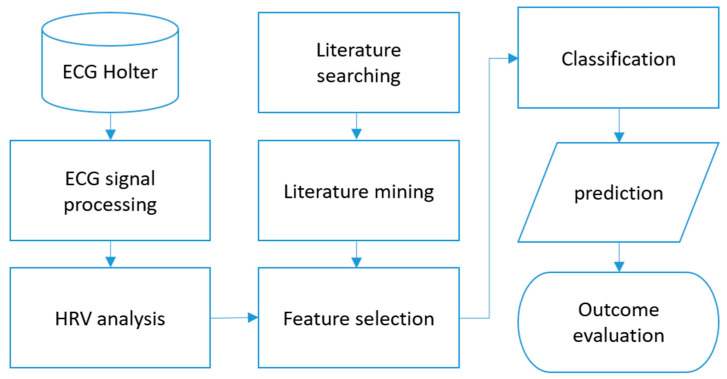
Flowchart of the study. ECG: Electrocardiogram, HRV: Heart rate variability. The ECG data are first filtered and signalized into a variety of common HRV parameters. HRV information is then extracted from the research literature, read by the large language model, and guided by us to identify the most appropriate HRV parameters. The parameters were then categorized to predict the occurrence of cardiovascular events. Finally, the effectiveness of the method was evaluated.

The data processing in this study was conducted at two ends, namely the literature review and ECG signal processing. The process flow is depicted in Figure 1. The ECG signals of the subjects were obtained from the public PhysioNet SHAREE database [13,32], which contains 24 h ECG recordings of 139 hypertensive patients obtained from the Holter database. Of these patients, 90 were male. The entire processing workflow—including literature retrieval, abstract interpretation, HRV parameter aggregation, ECG signal analysis, and prediction—was implemented through an end-to-end automated system using Python-based tools. This automated architecture ensured consistent application of the methodological logic and is outlined in Figure 1.

### 2.2. Literature Mining and Processing

We searched for papers containing HRV analysis parameters and vascular events by keyword search on PubMed, and then entered the returned papers into ChatGPT (GPT-3.5 API version) in a standardized format. The search was conducted using the following combined keywords: *(heart rate variability) AND ((Cerebrovascular Event) OR (Cardiovascular Event)) AND ((LF) OR (high frequency) OR (SDNN) OR (RMSSD))*. A Python 3.6-based web crawler was used to automate the retrieval of abstracts based on these search terms. Owing to the maximum word count input limit of 4096 on ChatGPT, only the abstract of each paper was used as the input. We then prompted ChatGPT to create a table for each paper based on the paper’s information. The table included the HRV parameters and the relationships of these parameters with the diagnoses/symptoms mentioned in the paper. This procedure can be regarded as a text-mining approach, where the LLM served as an automated tool to transform unstructured narrative abstracts into structured tabular data (parameter, directionality, and associated outcome). The HRV parameters in the table included common time domain analyses and frequency domain analyses, such as SDNN, rMSSD, VLF, LF, HF, ratio of low-to-high frequency power (LF/HF), and TP [11], which were selected based on the recommendation by Jarczok et al.

Example prompt: *“According to the article, please list LF, HF, LF/HF ratio, TP, VLF, SDNN, RMSSD which diseases/symptoms are related to each other? Does the value become higher or lower? If the item is not mentioned in the article, please write: N/A.*

Please present in a 3*8 table.

*notice:HF mean High frequency. NOT heart failure.*

*Form header: HRV Indicators | High/Low | Related Diseases/Symptoms”*

Here, ‘value’ refers to the reported direction of change in the HRV parameter. During this process, we observed that if an abstract did not mention specific HRV parameters or did not describe any related clinical symptoms or diagnoses, ChatGPT tended to return an empty table. We regarded these empty outputs as an indicator of irrelevance and excluded such papers from further consideration. The tables from all valid papers were compiled into a structured database, with metadata linking each table to its source article. The resulting collection allowed us to calculate the frequency and directional association (positive/negative) of each HRV parameter with vascular outcomes. This literature-derived dataset formed the Medical Literature Review Database (MLRD)—a central reference that served as the foundation for our subsequent feature selection framework. This literature mining and processing step was part of the broader automated pipeline. Details of the full system architecture can be seen in Figure 1.

### 2.3. Literature-Guided Feature Prioritization

Building upon the MLRD developed in the previous step, we implemented a novel feature selection approach that integrates ChatGPT as a literature-assisted summarization and consistency-checking tool to support evidence-based prioritization of HRV parameters reported to be associated with vascular events. This approach was grounded in the concept of Least-to-Most reasoning [29], which informed the overall design of our selection framework. Based on this concept, we developed the MLRD—a structured method unique to this study—for organizing and extracting HRV-related evidence from published literature.

The Least-to-Most prompting strategy is designed to enhance reasoning by decomposing complex tasks into simpler components that are solved in sequence, with each output used as context for the next. Unlike other prompting methods such as Chain-of-Thought, which often treat each step independently, Least-to-Most explicitly encourages cumulative reasoning. In this study, we adapted this strategy to mimic a typical literature-review progression: initially by identifying broad relational patterns, then by narrowing the set of relevant candidates, and finally by prioritizing features based on strength of supporting evidence. To minimize potential bias from pre-trained knowledge, we also included instructions prompting ChatGPT to perform reasoning before making any final decisions.

The MLRD was constructed to include, for each HRV parameter, the number of studies reporting positive and negative associations with vascular events. To prevent interference from ChatGPT’s prior training data, we anonymized the parameter names before inputting them into the model; for instance, HF, LF/HF, VLF, LF, TP, SDNN, and rMSSD were replaced with the labels A through G. Using this coded format and following the Least-to-Most framework, the feature selection process unfolded in three main phases. ChatGPT was first prompted to analyze the correlation patterns of each parameter by evaluating the consistency and directionality of their reported associations in the MLRD. This initial reasoning step allowed the model to identify which parameters exhibited meaningful and coherent relationships with vascular outcomes.

Example prompt: *“The following various parameters are substituted using the symbols: A B C D E F G. Please consider the consistency of the correlation and calculate the correlation between each parameter and the incidence rate.”*

Here, ‘correlation’ refers to the consistency of associations across studies, rather than statistical correlation coefficients. Based on this analysis, ChatGPT then screened out parameters with weaker or inconsistent correlations, retaining only those with relatively stronger relevance. At this point, the model was directed to further evaluate the remaining parameters by considering the volume of supporting literature, taking into account the number of studies reporting each association.

Example prompt: *“What are the parameters to be left for further investigation in terms of the strength or weakness of the correlation?”*

Finally, ChatGPT was prompted to rank the remaining parameters by the number of supporting studies.

Example prompt: *“Now that the number of documents is to be taken into account, choose the most relevant parameter from among these parameters, which one should be chosen?”*

This final consideration emphasized the statistical significance of parameter frequency across sources and helped ensure that parameters with both strong and well-supported correlations were prioritized.

**Figure 2:**
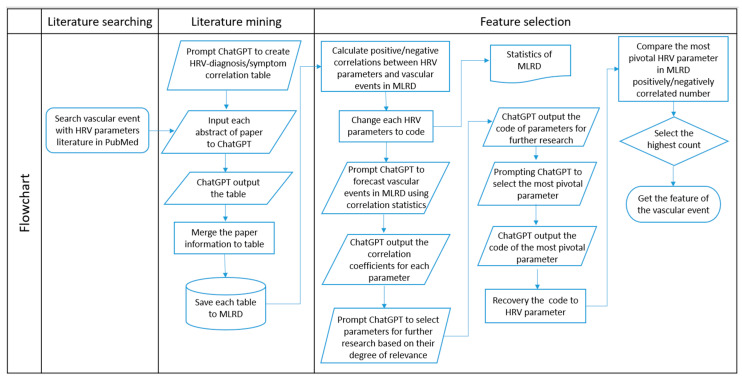
Flowchart of literature searching to feature selection. Ranking of the relevance of HRV parameters using a least-to-most prompt for the Chat Generative Pre-trained Transformer (ChatGPT). The decision-making process is divided into three concepts: determination of feature candidates, feature selection with correlation, and feature selection with the number of documents.

Once this reasoning sequence was complete, ChatGPT returned the anonymized code corresponding to the parameter prioritized through the predefined, stepwise reasoning procedure based on literature-derived evidence. To ensure reliability, the prompting procedure was repeated five times under identical conditions, and the outputs were consistent across runs. We translated this code back into the original HRV parameter and verified its selection by examining its prevalence across all recorded associations in the MLRD. The parameter with the highest combined count of positive and negative correlations was confirmed as the most pivotal. To conclude the process, we further examined the set of features associated with the vascular event corresponding to this key parameter, using this result to guide subsequent analysis within the study. Notably, this feature prioritization process does not constitute statistical feature selection or model optimization, but rather reflects a structured synthesis of reported evidence to guide subsequent analysis. The full workflow and prompting logic are visually summarized in Figure 2.

### 2.4. ECG Signal Processing and HRV Analysis

HRV analysis is not suitable for patients with arrhythmia, and patients with severe arrhythmia were excluded by expert interpretation. The steady-state ECG signals recorded during the daytime (08:00–16:00) were selected for 5 min analysis. The computer program used for HRV analysis herein was a modified version of our previous method [33]. In the QRS identification process, the computer starts by spotting all the peaks in the digital ECG signals, similar to general QRS detection algorithms. Then, it measures various parameters such as the height and duration of these peaks. These data help create standard QRS templates, represented by the average values and standard deviations (sigma) of these templates. Next, each QRS complex is recognized, and any ventricular premature complex or noise is filtered out based on how closely it matches the standard QRS templates. The R point within each valid QRS complex marks the timing of a heartbeat, and the gap between two consecutive R points (called the R–R interval) is calculated as the time between the current and the subsequent R points. To ensure nonparametric analysis, we performed frequency-domain analysis by using the Fast Fourier Transform (FFT). To ensure accuracy, we removed the direct current component and applied a Hamming window to mitigate side effects. The resulting power spectrum was then adjusted for any attenuation caused by the sampling process and the Hamming window. The power spectrum was quantified into various frequency-domain measurements as defined in [34] (VLF: 0.003–0.04 Hz; LF: 0.04–0.15 Hz; HF: 0.15–0.4 Hz). In addition, the LF and HF were subjected to natural logarithm transformation. The data distribution obtained as a result of these data-processing steps closely approximated a normal distribution.

### 2.5. Classification

**Figure 3:**
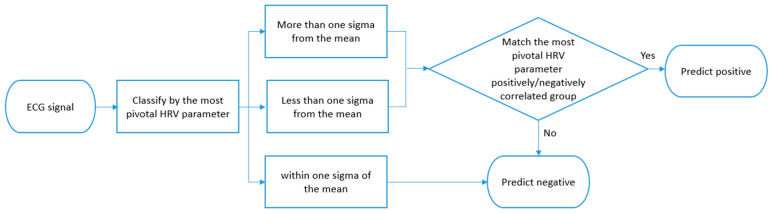
Classification by HRV distribution. ECG: Electrocardiogram. Cases were categorized into three groups based on the distribution of the most pivotal HRV parameter (above 1σ, within ±1σ, below 1σ). The directional association between each HRV parameter and vascular event risk was derived from the literature. A positive or negative outcome was predicted based on whether a patient’s HRV value was consistent with the literature-indicated risk direction.

Classification consisted of several steps, as illustrated in Figure 3. We first adopted the HRV parameters prioritized through the literature-guided reasoning process shown in Figure 2. For each selected HRV parameter, patients were categorized into three groups based on their distribution relative to the mean: (1) more than 1 standard deviation (σ) above the mean, (2) more than 1σ below the mean, and (3) within ±1σ of the mean. This stratification allowed us to identify patients with distinctly high or low HRV values in relation to the overall population. To determine the predicted outcome, we used the directional associations between HRV parameters and vascular event risk as extracted from the literature by the LLM. For example, if a parameter such as SDNN was frequently reported to be lower in patients with vascular events, then patients whose values were more than 1σ below the mean were considered to exhibit a risk-aligned pattern. A prediction was labeled as positive (i.e., event occurrence) when a patient’s HRV value was consistent with the literature-indicated risk direction. Conversely, when the patient’s HRV value contradicted the risk pattern found in the literature, the model predicted a negative (non-occurrence) outcome.

It is important to clarify that the terms “positive” or “negative correlation” used in this context do not refer to statistical correlation coefficients. Instead, they reflect qualitative, literature-derived associations indicating the direction of HRV changes linked to vascular risk.

### 2.6. Evaluation

The statistical tools we used to evaluate the vascular event prediction results are described as follows. The prediction results were plotted as a confusion matrix and evaluated for accuracy, sensitivity, and specificity. The confusion matrix contained four elements: true positives (TP), true negatives (TN), false positives (FP), and false negatives (FN). The definitions of accuracy, sensitivity (recall), and specificity are as follows.

Accuracy assesses overall correctness:

*Accuracy = (TP + TN)/(TP + TN + FP + FN)*

Sensitivity focuses on correctly identifying positives:

*Sensitivity = TP/(TP + FN)*

Specificity focuses on correctly identifying negatives:

*Specificity = TN/(TN + FP)*

The receiver operating characteristic (ROC) curve is a two-dimensional graph with a True Positive Rate (TPR) on the vertical axis and a False Positive Rate (FPR) on the horizontal axis. In the ROC curve, each point represents the TPR and FPR of the model at different thresholds. The upper left corner of the ROC curve indicates the best performance of the model as the TPR is maximum and the FPR is minimum at that point. As the threshold changes, the ROC curve bends from the lower left corner to the upper right. Next, we consider the area under the ROC curve (AUC), which provides a single value to quantify the predictive power of the model, usually between 0 and 1. The significance of AUC is that it represents the probability that the model will correctly categorize a positive case before a negative case when we randomly pick a positive case and a negative case. Therefore, the closer the AUC is to 1, the better the model’s performance is; conversely, the closer the AUC is to 0.5, the closer the model’s predictive power is to random guessing.

## 3. Results

After the exclusion of arrhythmia cases on the basis of expert interpretation, 114 cases of Holter ECG data remained. A total of 670 papers were retrieved by our keyword search. After reading the abstracts of these papers, ChatGPT was able to extract and summarize a table containing 538 positive and negative correlations of HRV parameters upon reviewing 140 abstracts for positive and negative correlation conditions. The statistical outcomes of these positive and negative correlations of HRV parameters are presented in Table 1. Because SDNN and rMSSD had no information extracted from the abstracts, no further analyses were performed subsequently.

**Figure 4:**
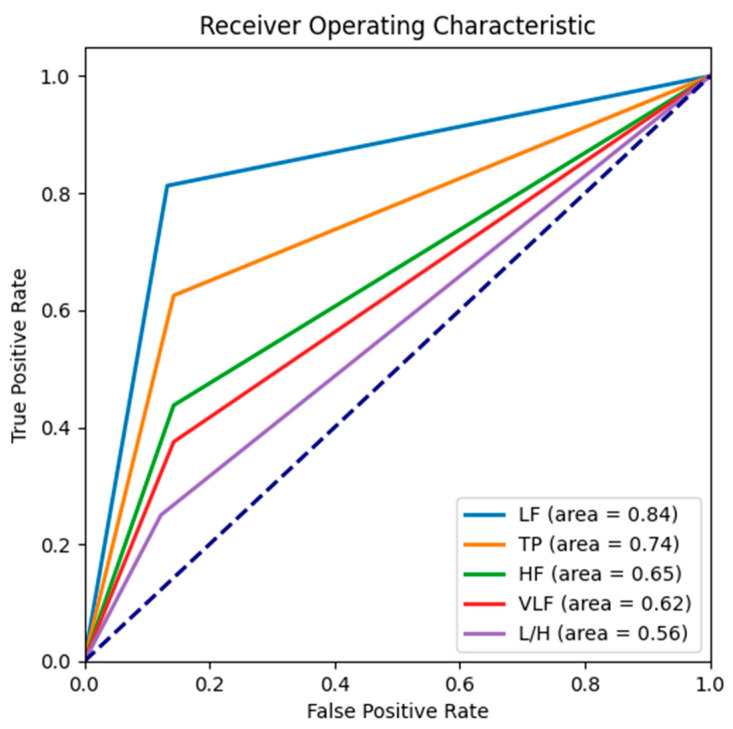
Receiver operating characteristic (ROC) curves and area Under the ROC curve (AUC) for different HRV parameters as predictive variables. The curve closest to the upper left corner is LF, and the AUCs calculated for the different parameters are, in descending order: LF, TP, HF, VLF, and L/H. It is shown that LF is the best classifier among these parameters. HF: Power in high-frequency range; LF/HF: Ratio of low-to-high-frequency power; VLF: Power in very-low-frequency range; LF: Power in low-frequency range; TP: Total power.

As shown in Figure 2, after disassembling the problem, the LLM-assisted literature analysis process retained codes C, D, and E. Then, in the last step, after considering the number of papers, only D was retained. D corresponds to LF. Therefore, LF was used as the literature-guided stratification parameter in subsequent steps. In the MLRD, there were more negative correlation tables in LF associated with vascular events, and therefore, the negative correlation group (Figure 3) was selected. An LF value that was less than one sigma away from the mean was used as the basis for categorization, according to the extracted literature evidence. To confirm that D was indeed the best choice, we additionally present the results of the confusion matrices, ROC and AUC of other parameters. In the confusion matrix of Table 2, LF has the highest number of true positive plus true negative number, indicating that it provides the most favorable classification performance ability among these parameters. Also, the ROC curve closest to the upper-left corner in Figure 4 is LF. The calculated AUC values for each parameter, listed from highest to lowest, are as follows: LF (0.84), TP (0.74), HF (0.65), VLF (0.62), and L/H (0.56). It is shown that LF is the best classifier among these parameters.

We computed the accuracy, sensitivity, and specificity of various HRV parameters separately, as presented in Table 3. Results indicate that LF demonstrates good performance in literature-guided vascular risk classification, with an accuracy of 86.3%, sensitivity of 81.3%, and specificity of 87.1%. TP exhibits an accuracy of 82.5%, sensitivity of 62.5%, and specificity of 85.7%. Its lower sensitivity suggests potential insensitivity in identification. HF shows an accuracy of 79.8%, sensitivity of 43.8%, and specificity of 85.7%. The lowest sensitivity of HF implies potential insensitivity to deviations from the normal range. LF/HF presents an accuracy of 78.9%, sensitivity of 25.0%, and specificity of 87.8%. The notably low sensitivity of this ratio may limit its clinical utility in diagnosis. VLF demonstrates an accuracy of 78.9%, sensitivity of 37.5%, and specificity of 85.7%. Its relatively lower sensitivity may affect its practicality in certain contexts. Overall, LF emerges as the suitable choice as a predictor.

To investigate potential sex differences, we further analyzed the performance of LF as a predictor separately for males and females. As shown in Table 4, among 49 female subjects, the mean ± SD of LF in the control group was 5.506 ± 1.149. The model achieved an accuracy of 84.6%, sensitivity of 87.5%, and specificity of 83.9%. In 90 male subjects, the mean ± SD of LF in the control group was 5.338 ± 1.078, with an accuracy of 86.3%, sensitivity of 87.5%, and specificity of 86.2%. These results suggest that the model performed consistently across sexes, with only minor differences in specificity. However, the relatively small number of female subjects limits the reliability of this sex-specific analysis, and further studies with larger balanced cohorts are warranted.

## 4. Discussion

This study explores the feasibility and effectiveness of using LLMs as part of a CDSS. Our approach resembles the process of human learning and application more closely than other machine learning algorithms. In addition, the greatest advantages of our method are the time saved on literature reading and the fact that past knowledge can be accumulated continuously in the MLRD. Overall, the adjustment speed of the proposed approach is faster than those of the other methods for continuous generation of up-to-date knowledge. This study unlocks the possibility of using LLMs as a CDSS in clinical settings. In the past, machine learning methods were used to categorize the same database with sensitivity and specificity rates of 71.4% and 87.8%, respectively, while our experimental results yielded sensitivity and specificity rates of 81% and 87%, respectively, underscoring the competitiveness of LLMs compared to traditional machine learning models.

In Table 5, we compare the accuracy, sensitivity, and specificity of the proposed approach with those of the approaches reported in other studies that used the same publicly available databases over the last three years. Although these studies are based on the same ECG database, direct comparisons should be interpreted with caution due to differences in dataset size, population characteristics, preprocessing methods, and experimental settings. The methods used in our study compared favorably with those used in the other studies, within 5%, in terms of prediction accuracy, except for decision trees and random under-sampling boosting [35]. However, our study’s sensitivity of 81.3% fell short of the other classifiers, with sensitivities exceeding 90%. Therefore, there may be a need for further optimization of our approach to correctly identify positive cases. Nevertheless, the proposed approach exhibited a specificity of 87.1%, which was higher than those of most of the other studies, indicating that our approach fared better in correctly identifying negative cases. Across literature-informed selection, model performance metrics, and external comparison, LF consistently outperformed other HRV parameters. Its strong and balanced predictive ability validates the effectiveness of our LLM-based framework and positions LF as a central feature for future applications in explainable, literature-driven vascular event prediction. Cardiovascular prediction results hold great reference value for clinicians.

In past large-scale public health surveys, the variance, VLF, LF, HF, and LF/HF distributions of HRV parameters were heavily skewed but were close to the normal distribution after natural logarithmic transformation [33]. Owing to differences in the groups and instruments used across the studies retrieved from PubMed, we did not use the data directly as the basis for subgrouping. Instead, we recalculated the means and standard deviations of the hypertensive groups in the database and then formed subgroups by using the sigma parameter. Although traditional machine learning methods have been shown to yield high classification performance in specific experimental settings [34,35,36], they often require extensive preprocessing, expert-defined feature selection, and retraining when new data becomes available. In contrast, our LLM-based approach supports continuous knowledge integration from the literature and allows for faster adaptation to evolving clinical contexts.

Currently, HRV CDSSs predominantly use algorithmic feature extraction, human-selected parameters, and machine learning models to categorize the results [39,40]. Our approach differs in that the classification parameters are selected by an LLM after reading the literature, which introduces an element of reliability. Unlike the Watson for Oncology expert system, we provide results from existing HRV examinations to guide physicians on which parameters to refer to for “predicting cardiovascular and cerebrovascular events,” rather than making clinical treatment recommendations.

The black box problem and its associated liability are significant challenges that must be solved before applying machine learning decision-making systems in clinical practice. Because there is no way to know if there is an unknown bias in a model, opaque decision logics remain difficult to adopt [41,42]. A few scientists have worked on dismantling the black-box approach to make the decision-making process transparent, thereby addressing the issue of liability [43,44,45]. Our study contributes to this effort by proposing a literature-grounded, traceable decision mechanism, in which interpretable HRV parameters are used to generate predictions and clusters are categorized using a statistical normal distribution. Each decision result can be traced back to a curated HRV evidence database, enabling physicians to verify the scientific rationale underlying each recommendation. This traceability enhances transparency and accountability, which are essential for clinical adoption. Most importantly, it helps reduce potential liability by ensuring that the system functions as a decision-support tool rooted in published literature, rather than as an opaque, autonomous decision-maker. Furthermore, unlike conventional machine learning pipelines that are difficult to update without retraining, our LLM-based method operates on a flexible, literature-driven framework. This enables clinicians to access up-to-date recommendations without needing specialized expertise in data science, enhancing clinical utility and scalability in fast-changing environments.

While our approach shows potential for real-world implementation, regulatory and infrastructural hurdles must be addressed. One such issue is data privacy. The use of commercial LLM APIs raises legitimate concerns regarding the protection of sensitive health information under regulations such as HIPAA. While no identifiable patient data were used in this study, future clinical deployment can adopt open-source LLMs hosted on secure, institution-controlled servers. This ensures that all data processing remains within hospital infrastructure, thereby mitigating the risk of sensitive information leakage and maintaining compliance with HIPAA or local equivalents.

In this study, the LF component demonstrated the most superior predictive performance for vascular events, a finding consistent with its role as an integrative marker of physiological adaptability and long-term health outcomes. Although the precise neuro-mechanical origins of LF remain a topic of active academic discussion, its clinical significance as a robust predictor is well-supported [46]. Specifically, LF power has been identified as a significant independent predictor of mortality and functional recovery in patients with cerebrovascular conditions, such as stroke [47]. This suggests that LF captures essential information regarding the body’s global regulatory capacity rather than isolated autonomic activity. Furthermore, the high predictive value of LF reflects the systemic integrity of autonomic control; as noted in long-term clinical observations, a diminished LF signal serves as a critical warning of homeostatic failure [48]. Therefore, by synthesizing evidence across these diverse clinical contexts, our LLM-based approach leverages LF as a high-fidelity ‘systemic health’ indicator, enabling the early detection of vascular dysregulation with greater sensitivity than traditional single-parameter metrics.

A least-to-most approach was applied for parameter selection in decision-making in this study. The methodology adopted herein considers the positive and negative correlations of the respective HRV parameters and the corresponding volumes of literature. The number of steps in the disentanglement problem depends on the type of parameters extracted from the statistical tables, where the number of steps increases as the number of parameters increases. We found that when ChatGPT was asked to make decisions without converting the HRV parameters to codes, it selected HF. If codes were used without problem solving, the parameter mentioned in the greatest number of studies was selected, regardless of the positive and negative correlations. The problem-splitting method allowed the LLM to make hierarchical decisions and identify the most indicative parameter, such as LF, from previous cardiovascular HRV studies.

To investigate potential sex differences, we briefly analyzed LF performance separately for females and males. The model achieved slightly higher accuracy and specificity in males (accuracy 86.3%, specificity 86.2%) than in females (accuracy 84.6%, specificity 83.9%), while sensitivity was the same (87.5%) across sexes. These small differences suggest that LF serves as a robust predictor for both sexes. However, the relatively small number of female subjects limits the reliability of this subgroup analysis, and further studies with larger, balanced cohorts are needed to confirm these observations.

Despite these strengths, several limitations must be acknowledged. The ECG dataset used for model validation consisted exclusively of patients with hypertension. Because hypertension is associated with altered cardiac electrophysiology and autonomic regulation, this disease-specific cohort may introduce selection bias and limit generalizability. Accordingly, the predictive outputs and HRV patterns identified in this study should be interpreted as primarily applicable to the hypertensive population. Validation in normotensive and more heterogeneous cohorts will be necessary to assess broader applicability. which introduces inherent limitations. A primary limitation of our approach is the observed 15% lower sensitivity compared with the highest-performing traditional classifiers, which reached sensitivities above 96%. With our model achieving 81% sensitivity, a substantial proportion of positive cases may be missed in real-world clinical settings. This gap currently restricts the clinical utility of the system, particularly in applications where early detection and risk stratification are critical. Addressing this limitation will require further optimization, such as expanding training datasets, incorporating additional HRV features, or combining literature-guided LLM reasoning with conventional machine learning methods to enhance detection of positive cases. Abstracts often prioritize main findings while omitting critical methodological details such as experimental design, statistical methods, patient demographics, inclusion/exclusion criteria, and measurement protocols. As a result, LLMs may miss context that is essential for accurate interpretation of HRV parameters. For example, the reported directionality or significance of a parameter in an abstract may not capture nuanced results or subgroup analyses available in the full text. This limitation could lead to underestimation or overestimation of the clinical relevance of certain HRV metrics, particularly those whose associations depend on patient-specific factors or complex statistical interactions. In our study, this was exemplified by SDNN and rMSSD (Table 1), which were excluded by the LLM due to insufficient extractable evidence in abstracts, despite their recognized importance in HRV research. Moreover, because abstracts tend to report only significant findings, parameters with non-significant associations may be underrepresented, introducing potential reporting bias. While abstract-based mining facilitates scalability and efficiency, future work incorporating full-text analysis is needed to capture richer context, reduce reporting bias, and improve the reliability of LLM-guided HRV interpretation.

In this study, we excluded ECG recordings with arrhythmias to ensure the clarity and reliability of HRV parameter extraction. While this approach facilitated model training and validation under controlled conditions, it may limit the generalizability and robustness of the model in real-world clinical settings, where arrhythmic events are common. Consequently, the current model may perform less reliably when encountering noisy or irregular signals that were not represented in the training data.

Future work could explore alternative strategies to incorporate arrhythmic data without compromising model performance. Possible approaches include: (1) augmenting the dataset with labeled arrhythmic segments and training the model to recognize and appropriately weight these signals, (2) applying preprocessing or signal-cleaning techniques to mitigate noise while retaining clinically relevant arrhythmia patterns, and (3) developing hybrid models that combine HRV-based features with arrhythmia-specific metrics to enhance predictive accuracy under variable conditions. Incorporating such strategies would improve the robustness and clinical applicability of the CDSS, making it more reflective of real-world patient populations.

## 5. Conclusions

In conclusion, the study demonstrates that LF is the most effective HRV parameter for predicting vascular events, outperforming other parameters in accuracy, sensitivity, and specificity. The results were consistent across five repeated runs, indicating the stability and reliability of the proposed approach. Performance analysis by sex showed similar predictive outcomes for both males and females, although the smaller female sample warrants cautious interpretation. These findings validate the effectiveness of the LLM-based framework in literature-informed parameter selection and reliable vascular event prediction. Overall, LF is confirmed as a robust and evidence-supported predictor, highlighting its central role in explainable, data-driven cardiovascular risk assessment. In summary, this study presents a feasibility-oriented framework demonstrating how LLM-assisted literature synthesis can support transparent, literature-guided HRV stratification for vascular risk assessment.

## Figures and Tables

**Table 1:** Positive and negative correlations of HRV parameters to vascular events extracted from the abstracts of 140 papers. When these HRV parameters were imported into our model, they were converted into code for subsequent analysis.

HRV Parameters and Code		Cardiovascular and Cerebrovascular Event	
		Positively Correlated	Negatively Correlated
HF	A	104	118
LF/HF	B	57	88
VLF	C	6	24
LF	D	29	84
TP	E	7	21
SDNN	F	0	0
rMSSD	G	0	0

HF: Power in high-frequency range; LF/HF: Ratio of low-to-high-frequency power; VLF: Power in very-low-frequency range; LF: Power in low-frequency range; TP: Total power; SDNN: Standard deviation of all normal-to-normal beat intervals; rMSSD: Square root of the mean of the square of successive normal-to-normal beat intervals.

**Table 2:** The confusion matrix results of the study participants’ data using various HRV parameters as predictor variables. Confusion matrices for HF, LF/HF, VLF, LF, and TP are provided. * The results indicate that LF demonstrates the best performance.

			True Condition	
			Positive	Negative
Prediction outcome	HF	Positive	7	9
		Negative	14	84
	LF/HF	Positive	4	12
		Negative	12	86
	VLF	Positive	6	14
		Negative	10	84
	* LF	Positive	13	13
		Negative	3	85
	TP	Positive	10	14
		Negative	6	84

HF: Power in high-frequency range; LF/HF: Ratio of low-to-high-frequency power; VLF: Power in very-low-frequency range; LF: Power in low-frequency range; TP: Total power.

**Table 3:** The accuracy, sensitivity, and specificity of different HRV parameters as predictive variables. LF exhibits the best performance in both accuracy and sensitivity. While in terms of Specificity, LF ranks just below LF/HF, with a negligible difference.

HRV Parameters	Accuracy %	Sensitivity %	Specificity %
LF	86.3	81.3	87.1
TP	82.5	62.5	85.7
HF	79.8	43.8	85.7
LF/HF	78.9	25.0	87.8
VLF	78.9	37.5	85.7

HF: Power in the high-frequency range; LF/HF: Ratio of low-to-high-frequency power; VLF: Power in the very-low-frequency range; LF: Power in the low-frequency range; TP: Total power.

**Table 4:** Performance of LF as a predictor of vascular events stratified by sex. Accuracy, sensitivity, and specificity are reported separately for male (*n* = 74) and female (*n* = 40) subjects.

Gender	Accuracy %	Sensitivity %	Specificity %
Female	84.6	87.5	83.9
Male	86.3	87.5	86.2

**Table 5:** Comparison of accuracy, sensitivity, and specificity of studies using HRV from the same electrocardiogram (ECG) database to predict cardiovascular or cardiovascular and cerebrovascular events.

Classifier	Accuracy %	Sensitivity %	Specificity %	Year [Reference]
Large language model with least-to-most prompt	86.3	81.3	87.1	This study
Random forest	85.7	71.4	87.8	2015 [13]
Decision trees and random under-sampling boosting	97.1	92.9	97.6	2020 [35]
Support vector machines	91.8	96.7	87.1	2022 [36]
Deep neural networks	90.2	95.8	85.2	
Extreme gradient boosting	89.1	93.8	85.1	
Adaptive boosting	89.5	94.6	84.9	2022 [37]
Majority-vote over multiple ECG segments for risk assessment	78.0	90.0	67.0	2021 [38]

**Note:** Although the studies listed in this table are based on the same publicly available ECG database, direct quantitative comparisons should be interpreted with caution due to differences in dataset size, population characteristics, preprocessing strategies, event definitions, and experimental protocols across studies.

## Data Availability

The datasets analyzed during the current study are available in the PhysioNet repository, https://physionet.org/content/shareedb/1.0.0/ (accessed on 5 May 2023).

## Abbreviations

The following abbreviations are used in this manuscript:

HRV	Heart rate variability
ECG	Electrocardiogram
HF	Power in high-frequency range
LF/HF	Ratio of low-to-high-frequency power
VLF	Power in very-low-frequency range
LF	Power in low-frequency range
TP	Total power
SDNN	Standard deviation of all normal-to-normal beat intervals
rMSSD	Square root of the mean of the square of successive normal-to-normal beat intervals
NLP	natural language processing
LLM	large language model
COT	chain-of-thought
ANS	autonomic nervous system
CDSS	Clinical Decision Support System
MLRD	Medical Literature Review Database
